# MicroRNA-206 overexpression promotes apoptosis, induces cell cycle arrest and inhibits the migration of human hepatocellular carcinoma HepG2 cells

**DOI:** 10.3892/ijmm.2014.1800

**Published:** 2014-06-11

**Authors:** WEIWEI LIU, CHUANMING XU, HUIFANG WAN, CHUNJU LIU, CAN WEN, HONGFEI LU, FUSHENG WAN

**Affiliations:** 1 Department of Biochemistry and Molecular Biology, Basic Medical College of Nanchang University, Nanchang, Jiangxi 330006, P.R. China; 2 Medical Experiment Education Department of Nanchang University, Nanchang, Jiangxi 330006, P.R. China; 3 Department of Clinical Laboratory, The Affiliated Hospital of Jiangxi College of Chinese Medicine, Nanchang, Jiangxi 330006, P.R. China

**Keywords:** hepatocellular carcinoma, microRNA-206, apoptosis, cell cycle arrest, migration, tumor suppressor

## Abstract

MicroRNA-206 (miR-206) is known to regulate cell proliferation and migration and is involved in various types of cancer. However, the role of miR-206 in human hepatocellular carcinoma (HHC) has not been previously reported. In the present study, the expression of Notch3 in HCC and adjacent non-neoplastic tissue was immunohistochemically assessed on formalin-fixed, paraffin-embedded sections. miR-206 mimics were transiently transfected into HepG2 cells using Lipofectamine™ 2000. Subsequently, we evaluated the role of miR-206 in cell proliferation, apoptosis, cell cycle arrest and migration by MTS assay, Hoechst 33342 staining, Annexin V-FITC/PI assay, flow cytometry and wound healing assay. Using quantitative reverse transcription polymerase chain reaction (qRT-PCR) and western blot analysis, we detected the expression of Notch3, Bax, Bcl-2, Hes1, p57 and matrix metalloproteinase (MMP)-9 at the mRNA and protein level, respectively. In addition, we measured the expression of miR-206 at the mRNA level and that of caspase-3 at the protein level. After miR-206 was upregulated in HepG2 cells, Notch3, Hes1, Bcl-2 and MMP-9 were downregulated both at the mRNA and protein level, whereas p57 and Bax were upregulated. Cleaved caspase-3 protein expression was also markedly increased. Cell proliferation was significantly attenuated and apoptosis was markedly increased. Furthermore, miR-206 overexpression induced cell cycle arrest and inhibited the migration of HepG2 cells. Taken together, our results uggest that miR-206 is a potential regulator of apoptosis, the cell cycle and migration in HepG2 cells and that it has the potential for use in the targeted therapy of HCC and is a novel tumor suppressor.

## Introduction

Hepatocellular carcinoma (HCC) represents the fifth most common and aggressive malignancy worldwide and the third in terms of mortality (1,2). HCC has become a serious threat to human health due to its rising incidence and high metastatic recurrence and mortality rates (3,4). Although patients diagnosed with this malignant disease can benefit from some of the existing effective treatments, including liver transplantation, surgical resection, embolization, stereotactic body radiation therapy, ablation and chemotherapy, which improve their chances of survival (5), the current treatment options cannot smeet the requirements for the survival of HCC patients and the prognosis remains dismal (6). Therefore, the search for novel and more effective treatment strategies is of particularl importance. Gene therapy, which is increasingly being tested in clinical trials and has shown potential in clinical practice, has the advantage of high specificity, efficiency and security (7), and has been shown to have potential future perspectives (8).

miR-206, a member of the muscle-specific miR-1 family of muscle-specific microRNAs (myomiRs), is a skeletal muscle-specific miRNA involved in muscle development (9). However, studies have revealed that miR-206 is closely related to various tumors. The ectopic expression of miR-206 has been shown to inhibit the growth of rhabdomyosarcoma (RMS) (10), breast cancer (11,12), endometrial endometrioid carcinoma (EEC) (13), lung cancer (14) and HeLa cells (15). Furthermore, fluorescence-activated cell sorting (FACS) has demonstrated that miR-206 activates apoptosis in lung cancer (14) and HeLa cells (15) and induces cell cycle arrest at the G0/G1 phase of the cell cycle in RMS (10) and EEC cells (13). Cell invasive and migratory ability has also been shown to be impaired by miR-206 in RMS (10), EEC (13), lung cancer (14) and HeLa cells (15).

Although the multiple anticancer functions of miR-206 have been confirmed, its underlying anticancer mechanisms of action are not yet fully understood. However, it is a worth noting that Song *et al* first identified an almost perfect complementarity between miR-206 and the 3′-untranslated regions (3′-UTRs) of both mouse and human Notch3 and found that the ectopic expression of miR-206 induced apoptotic cell death in HeLa cells, which was associated with its inhibition of Notch3 signaling (15). Early research has demonstrated that the Notch3 receptor, one of the mammalian Notch family receptors (Notch1-4), plays an important role in cellular differentiation (16) and embryonic development (17). Of note, a growing body of evidence in recent years has indicated that Notch3 is also involved in the regulation of cancer development and progression (18–22). Using immunohistochemistry, Zhou *et al* demonstrated that Notch3 had a stronger positive degree of expression in lung squamous cell carcinoma and adenocarcinoma compared with the corresponding non-tumor tissue (P<0.01) (23). Moreover, Notch3 overexpression has been shown to significantly correlate with poor prognosis in human non-small cell lung cancer (NSCLC) (24). By contrast, the inhibition of Notch3 by γ-secretase inhibitor (GSI) induces apoptosis and suppresses the proliferation of cancer cells through the downregulation of the pro-survival proteins, pBcl-2 and pBcl-xL, and not Bax in NSCLC (25). A decrease in Notch3 expression can also activate apoptosis by increasing the cleavage of caspase-3 and poly(ADP-ribose) polymerase (PARP) (21).

Moreover, an increasing number of studies has indicated that Notch3 contributes to the promotion of HCC development and progression. Notch3, Jagged1, Delta1 and the downstream effector gene, hairy and enhancer of split 1 (Hes1), are highly expressed in the HepG2 tumor cell line, which was thought to be necessary for malignant liver cell proliferation (19). In addition, by regulating matrix metalloproteinase (MMP)-2 and MMP-9 through the ERK1/2 pathway, high Notch3 expression also strongly correlates with HCC metastasis (26). However, the downregulation of Notch3 in 2 HCC cell lines has been shown to result in the downregulation of Hes1, the upregulation of CDKN1C/p57, and reduced cell growth through the induction of senescence instead of apoptosis (27).

In this study, we aimed to investigate the potential function of miR-206 in the development and progression of HCC. It was hypothesized that Notch3 is a direct target gene of miR-206 in HCC cells. miR-206 mimics were transiently transfected into HepG2 cells. We found that miR-206 significantly suppressed tumor growth and metastasis at least in part by targeting the Notch3 signaling pathway *in vitro*. To the best of our knowledge, this study is the first to reveal the function and possible underlying mechanisms of action of miR-206 in HCC and suggests that miR-206 has the potential for use in the targeted therapy of HCC.

## Materials and methods

### Immunohistochemistry and evaluation of immunostaining

Formalin-fixed and paraffin-embedded (FFPE) tissue samples from HCC and adjacent non-neoplastic tissues (at least 1.5 cm away from the tumor) were collected from 12 patients who were histopathologically diagnosed with primary HCC and had undergone surgical treatment at the Second Affiliated Hospital of Nanchang University (Nanchang, China) in the last 5 years. Sections (4-μm-thick) mounted on glass slides were processed for immunohistochemistry. All slides were dewaxed in xylene and dehydrated in an alcohol gradient; endogenous peroxidase activity was quenched with 3% hydrogen peroxide for 10 min. Antigen retrieval was obtained by heating the slides covered with citrate buffer (pH 6.0) at 95°C for 10 min. Subsequently, 10% goat serum albumin was used to block non-specific binding by incubating the sections for 1 h at room temperature. Gently tilting without washing, the sections were then incubated with anti-Notch3 (1:50 dilution; polyclonal anti-Notch3, M-134: sc-5593; Santa Cruz Biotechnology, Inc., Santa Cruz, CA, USA) diluted in 1× TBS for 2 h in a moist box at room temperature. The sections were then incubated with the secondary antibody at room temperature for 1 h and rinsed in phosphate-buffered saline (PBS). Diaminobenzidine (DAB) was used as the chromogen and the sections were counterstained with hematoxylin. For negative controls, the sections incubated with PBS instead of the primary antibody. Brown particles present in the cytoplasm and/or nuclei were considered positive. Imaging analysis was conducted under a microscope (Olympus, Tokyo, Japan); the exact location of the measured visual field was determined and then 3 complete visual fields without overlap were randomly selected for viewing. A validated semi-quantitative scale was used to assess the immunostaining: ‘−’ denotes no hepatocyte staining; ‘+/−’ denotes occasional weak hepatocyte staining; ‘+’ denotes <5% hepatocyte staining; ‘++’ denotes 5–30% hepatocyte staining; and ‘+++’ denotes >30% hepatocyte staining.

### Cell culture and transfection

HepG2 cells were grown in Dulbecco’s modified Eagle’s medium (DMEM) (Solarbio, Beijing, China) plus 10% fetal calf serum (FBS; TransGen Biotech, Beijing, China), 2 mM L-glutamine, 100 U/ml penicillin and 100 μg/ml streptomycin (all reagents were from Gibco-BRL Life Technologies, Gaithersburg, MD, USA) and incubated in a 5% CO_2_ humidified incubator at 37°C. Cy3-modified miR-206 mimic and Cy3-modified mimic negative control were purchased from RiboBio Co., Ltd. (Guangzhou, China). For convenience, Cy3-modified miR-206 mimic and Cy3-modified mimic negative control were simply referred to as miR-206 and negative control (NC), respectively. Complete medium without antibiotics was used to culture the cells at least 24 h prior to transfection. The cells were washed with PBS and then transiently transfected with 100 nM miR-206 or NC using Lipofectamine™ 2000 (Invitrogen, Carlsbad, CA, USA) according to the manufacturer’s instructions.

### Cellular proliferation assay

The HepG2 cells (1,000 cells/well) were seeded in a 96-well plate and incubated under normal culture conditions for 24 h prior to transfection. Cell proliferation was measured using the CellTiter 96^®^ AQueous One Solution Cell Proliferation Assay (MTS) kit (Promega, Beijing, China) according to the manufacturer’s instructions. MTS reagent (20 μl) was added to the cells in each well followed by incubation for 2 h in a 37°C, 5% CO_2_ humidified incubator at 0, 24, 48, 72 and 96 h after transfection. The absorbance (A) of each plate was measured at 490 nm.

### Hoechst 33342 staining

The HepG2 cells were plated in 12-well plates at a density of 1×10^5^ cells/well 1 day prior to transfection. Forty-eight hours after transfection, the plates were washed twice with PBS, then 500 μl Hoechst 33342 (Beyotime, Shanghai, China) were added to each well followed by incubation for 30 min at 37°C in the dark. Nuclear DNA staining was observed using a fluorescence microscope (Olympus). A total of 500 cells was counted from 5 random high-power fields and the fluorescence staining percentage of positive cells was expressed as the ratio of apoptotic cells with respect to the total amount of cells.

### RNA isolation and quantitative reverse transcription polymerase chain reaction (qRT-PCR)

Total RNA from the cultured cells was extracted using TRIzol reagent (TransGen Biotech, Beijing, China) according to the manufacturer’s instructions. miRNA levels were measured by qRT-PCR. For the qRT-PCR detection of mature miR-206 expression, we purchased the Bulge-Loop™ miRNA qRT-PCR Primer Set and the miRNA qRT-PCR Control Primer Set (both from RiboBio). RNA (1 μg) was converted into cDNA using the PrimeScript™ RT reagent kit with gDNA Eraser (Takara, Dalian, China) according to the manufacturer’s instructions. qRT-PCR was performed using SYBR^®^ Premix Ex Taq™ II (Takara) in the ABI PRISM^®^ 7500 real-time PCR system (Applied Biosystems, Foster City, CA, USA). β-actin and U6 were used as endogenous controls. In addition, melting curves were used to evaluate non-specific amplification. The relative expression level was calculated using the ΔΔCt method. The primer sequences used in this study are presented in Table I.

### Western blot analysis

Forty-eight hours after transfection, total protein was extracted from the HepG2 cells using RIPA cell lysis reagent containing proteinase and phosphatase inhibitors (Solarbio) at 4°C for 30 min. Cell lysates were centrifuged at 12,000 × g for 20 min at 4°C, and the protein concentrations of the supernatants were determined using the BCA protein assay reagent kit (Beyotime). The supernatants containing total protein were then mixed with a corresponding volume of 5× SDS loading buffer and heated at 95°C for 5 min; 40 μg of total protein from each sample were concentrated on 5% Tris-glycine SDS gels, separated on 12% Tris-glycine SDS gels and transferred onto 0.22-μm polyvinylidene fluoride (PVDF) membranes. The membranes were blocked with 5% non-fat dry milk in TBST and incubated overnight with the appropriate primary antibody. The primary antibodies and dilutions used were as follows: anti-Notch3 (Cat. no. 5276, 1:200), anti-p57 (Cat. no. 2557, 1:500), anti-MMP-9 (Cat. no. 852, 1:200), anti-caspase-3 (Cat. no. 9662, 1:300) purchased from Cell Signaling Technology (Beverly, MA, USA), anti-Hes1 (ab71559, 1:300; Abcam), anti-Bax (Cat. no. 50599-2-Ig; 1:500) and anti-Bcl-2 (Cat. no. 12789-1-AP, 1:500), both from ProteinTech. The membranes were then incubated with the secondary horseradish peroxidase-conjugated AffiniPure goat anti-rabbit lgG (H+L) (1:1,000; TransGen Biotech) or the secondary horseradish peroxidase-conjugated AffiniPure goat anti-mouse lgG (H+L) (1:1,000; ZSGB-BIO, Beijing, China). Anti-β-actin monoclonal antibody (1:1,000; ZSGB-BIO) was used as an endogenous control. The quantification of western blot analyses was performed using Quality One 4.6.2 software, and the integral optical density (IOD) of each band was determined. The relative protein level was used to evaluate the differences in protein expression between the miR-206 -treated group and the NC group; the relative protein level = (IOD ratio between the target gene product bands and the β-actin protein bands in the miR-206-treated group)/(IOD ratio between the target gene product bands and the β-actin protein bands in the NC group).

### Annexin V-FITC/PI analysis

The HepG2 cells were harvested at 48 h after transfection. Cell apoptosis was detected using an Annexin V-FITC/PI apoptosis detection kit (BestBio, Shanghai, China) following the manufacturer’s instructions, and the percentage of apoptotic cells was calculated using a Beckman Coulter FACSCalibur flow cytometer (Beckman Coulter, Inc., Fullerton, CA, USA).

### Cell cycle analysis

The HepG2 cells were collected at 48 h after transfection and fixed with 70% ethanol in PBS at −20°C overnight. Cell cycle analysis was performed using the cell cycle kit (BestBio) according to the manufacturer’s specifications, and cell cycle distribution was analyzed using a Beckman Coulter FACSCalibur flow cytometer (Beckman Coulter, Inc.).

### Wound healing assay in vitro

The HepG2 cells were seeded in 6-well plates and incubated for 24 h; a linear wound was tehn created by dragging a 1-ml pipette tip through the monolayer prior to transfection. Cellular debris was removed by gentle washes with culture medium, following which transfection was performed immediately, and the cells were allowed to migrate for a further 24 h. The healing process was dynamically photographed after the wound was introduced using a microscope (Olympus). The gap size was analyzed using Image-Pro Plus 6.0 software. The residual gap between the migrating cells from the opposing wound edge was expressed as a percentage of the initial gap size.

### Statistical analysis

All experiments were repeated 3 times independently. The results are presented as the means ± standard deviation (SD). A rwo-tailed paired t-test was performed using SPSS 19.0 software in order to detect significant differences in measured variables between groups. A value of P<0.05 was considered to indicate a statistically significant difference.

## Results

### Differential expression of Notch3 in HCC and adjacent non-neoplastic tissues

**Figure 1:**
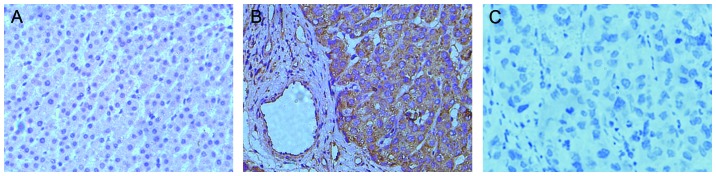
Notch3 expression in the cytoplasm of hepatocytes in hepatocellular carcinoma (HCC) and adjacent non-neoplastic tissues [immunohistochemistry (IHC); magnification, ×400]. (A) Adjacent non-neoplastic tissue: occasional weak staining is not evident in the cytoplasm. (B) HCC: strong positive staining is visible in the cytoplasm. (C) Negative control.

Immunostaining revealed a high Notch3 protein expression in the cytoplasm of the neoplastic hepatocytes in 12 out of the 12 (100%) HCC samples compared with occasional weak hepatocytic staining in their corresponding adjacent non-neoplastic tissue samples. Representative immunostaining patterns of the Notch3 expression are shown in Fig. 1.

### Inhibition of cell proliferation following transfection with miR-206

**Figure 2:**
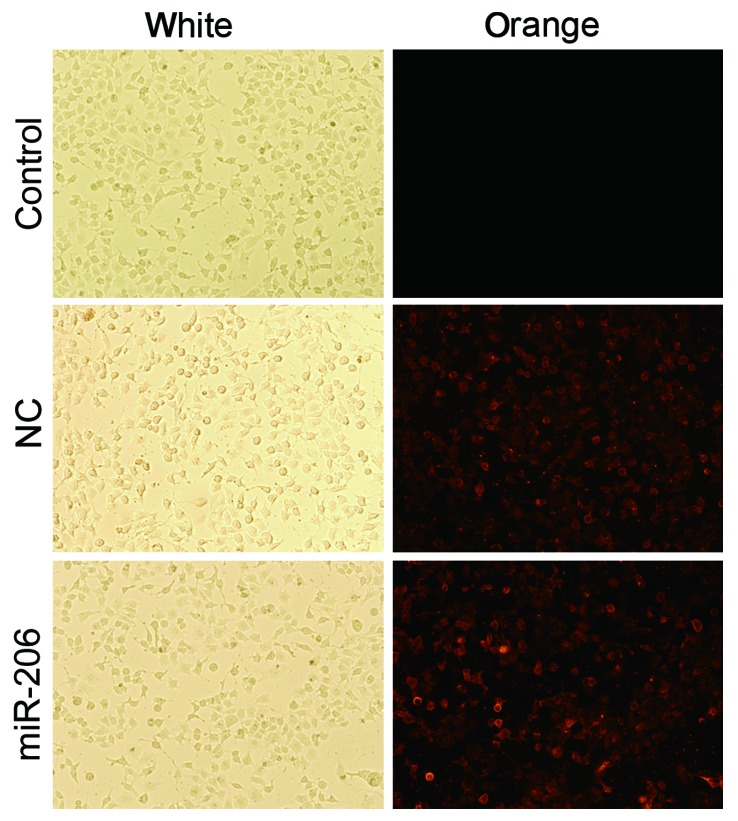
Effectiveness of transfection of HepG2 cells with miR-206 mimic or negative contorl (NC). Magnification, ×200. White, white light used as the excitation light; orange, green light used as the excitation light.

**Figure 3:**
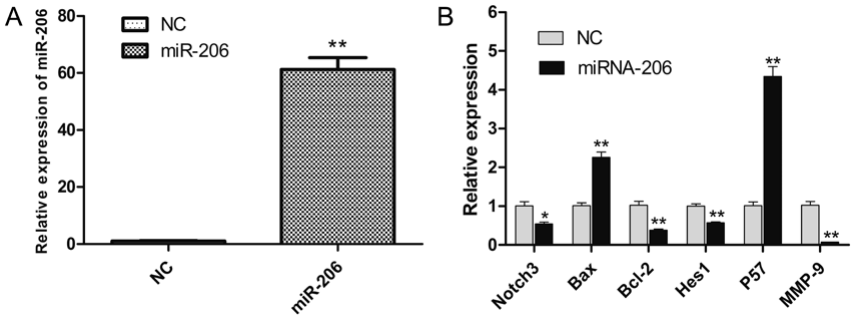
qRT-PCR analysis of miR-206, Notch3, Bax, Bcl-2, Hes1, p57 and matrix metalloproteinase-9 (MMP-9) mRNA expression in each group of HepG2 cells. (A) miRNA-206 relative expression. (B) Notch3, Bax, Bcl-2, Hes1, p57 and MMP-9 mRNA relative expression. ^*^P<0.05 and ^**^P<0.01.

**Figure 4:**
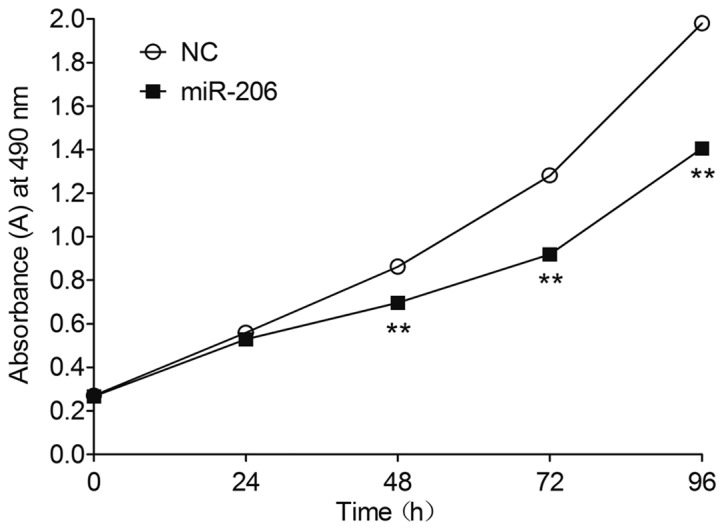
Elevated miR-206 expresion inhibits the proliferation of HepG2 cells. Cell proliferation was determined by MTS assay. ^**^P<0.01.

To investigate the functional role of miR-206, Cy3-modified miR-206 mimic and Cy3-modified mimic negative control were successfully transiently transfected into the HepG2 cells (Fig. 2). Furthermore, the mRNA levels of miR-206 were analyzed by qRT-PCR. We found that the miR-206 mimic-treated cells had an approximately 60-fold greater expression of mature miR-206 than the cells transfected with the negative control mimic (Fig. 3A). Cell proliferation was significantly decreased in the cells following 48 h of transfection with miR-206 (Fig. 4). These results indicate that miR-206 overexpression decreases the proliferation of human hepatocellular carcinoma HepG2 cells.

### miR-206 overexpression promotes apoptotic cell death in HepG2 cells

**Figure 5:**
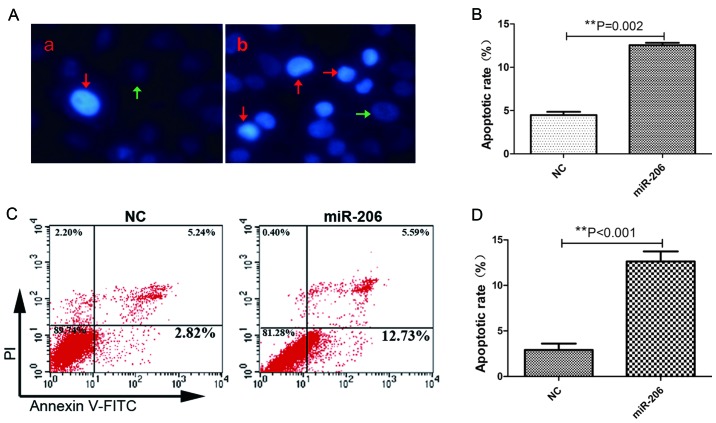
Overexpression of miR-206 promotes apoptosis of human hepatocellular carcinoma HepG2 cells. (A) Hoechst 33342 staining of each group of HepG2 cells. (a) Mimic negative control (NC) group. (b) miRNA-206-transfected group. The red and green arrows indicate the apoptotic and normal cells, respectively. Magnification, ×200. (B) Hoechst 33342 staining of apoptotic rate in each group of HepG2 cells. (C) Annexin V-FITC/PI staining and flow cytometric analysis of cell death. The lower right quadrant of each plot indicates early apoptotic cells. (D) FACS analysis of apoptotic rate in each group of HepG2 cells. ^**^P<0.01.

**Figure 6:**
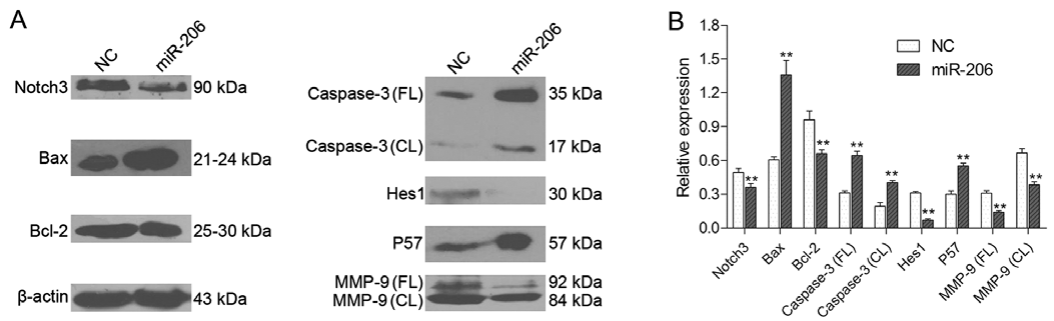
Western blot analysis of Notch3, Bax, Bcl-2, caspase-3, Hes1, p57 and matrix metalloproteinase-9 (MMP-9) expression in each group of HepG2 cells. (A) A representative western blot is shown. (B) Relative protein expression levels of Notch3, Bax, Bcl-2, full length (FL) caspase-3, cleaved (CL) caspase-3, Hes1, p57, MMP-9 FL and MMP-9 CL were assessed calculating the integral optical density (IOD)-values. IOD values were normalized to those of β-actin protein. ^**^P<0.01.

Decreased apoptotic activity in HCC cells is one of the most important features of HCC (28). In this study, in order to investigate whether miR-206 induces cellular apoptosis, Hoechst 33342 staining and Annexin V-FITC/PI flow cytometry were conducted. The miR-206-treated group showed increased numbers of Hoechst 33342 positively stained cells 48 h after transfection, indicating an enhanced apoptotic activity (Fig. 5A–b). In accordance with Hoechst 33342 staining, FACS analysis further confirmed that the cells transfected with miR-206 underwent more apoptosis compared with the miR-206 mimic-treated cells (Fig. 5C). There was an approximately 2.0- or 3.0-fold increase in the percentage of apoptotic cells in the HepG2 cells overexpressing miR-206 (Fig. 5B and D). Moreover, qRT-PCR analysis revealed that the relative expression of Notch3 and Bcl-2 was markedly reduced, whereas that of Bax was increased at the mRNA level following transfection with miR-206 (Fig. 3B). In addition, miR-206 overexpression downregulated Notch3 and Bcl-2 expression and upregulated Bax and caspase-3 expresssion at the protein level, as shown by western blot analysis (Fig. 6); the protein expression of cleaved caspase-3 (caspase-3 CL) in particular was markedly increased. Of note, these data support our hypothesis that Notch3 is likely to be a direct target gene of miR-206 in HepG2 cells. Furthermore, these results indicate that the pro-apoptotic effect of miR-206 in HepG2 cells is at least partially dependent on Notch3-mediated mitochondrial apoptotic signaling.

### miR-206 induces cell cycle arrest in HepG2 cells

**Figure 7:**
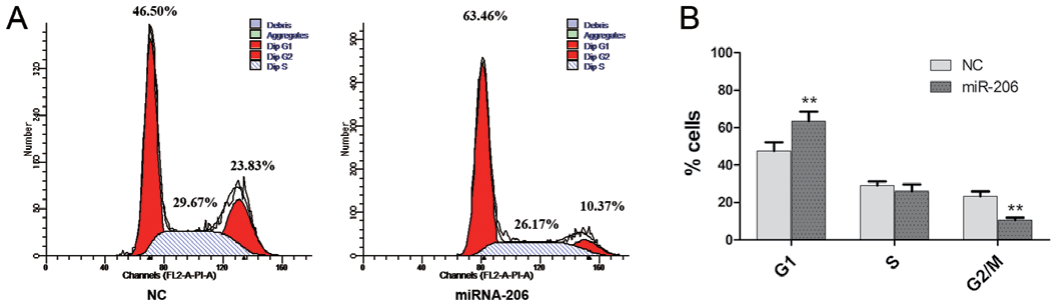
miR-206 induces cell cycle arrest of HepG2 cells. (A) Mimic negative control *NC)- and miR-206-transfected cells were subjected to FACS analysis. Representative FACS analysis images are shown. Cell cycle distribution was as follows: NC-transfected cells (G1 46.50%; S 29.67%; G2/M 23.83%), miR-206-transfected cells (G1 63.46%; S 26.17%; G2/M 10.37%). (B) Proportions of cells in the G0/G1 and G2/M phases exhibited significant differences between the 2 groups. ^**^P<0.01.

Flow cytometry was used to investigate the effects of miR-206 on the cell cycle. Our results revealed that the proportion of the cells in the G0/G1 and G2 phases was did not altered in the NC group. By contrast, the transfection of HepG2 cells with miR-206 resulted in an accumulation of cells in the G0/G1 phase and a decrease in the number of cells in the G2/M phase compared with the NC group. Flow cytometric analysis indicated that miR-206 overexpression slowed down cell cycle progression and caused cell cycle G1 phase blockage in the HepG2 cells (Fig. 7A). The proportion of cells in the G0/G1 and G2/M phases exhibited significant differences between the 2 groups (Fig. 7B). In order to discover the probable underlying mechanisms of action of miR-206 in inducing cell cycle arrest, we further analyzed the expression of Hes1 and p57 in HepG2 cells at the mRNA and protein level. A significant inverse correlation between Hes1 and p57 expression was demonstrated by qRT-PCR (Fig. 3B) and western blot analysis (Fig. 6), suggesting that Hes1 participates in regulating p57 mRNA transcription in the HepG2 cells. Moreover, Hes1 is acknowledged to be a direct target gene of Notch3. Taken together, our results indicate that the effects of miR-206 on the cell cycle (causing cell cycle arrest) are possibly mediated through the crosstalk between these 3 genes (Notch3-Hes1-p57 signaling) in the HepG2 cells.

### Cellular migration is impaired following transfection with miR-206 in HepG2 cells

**Figure 8:**
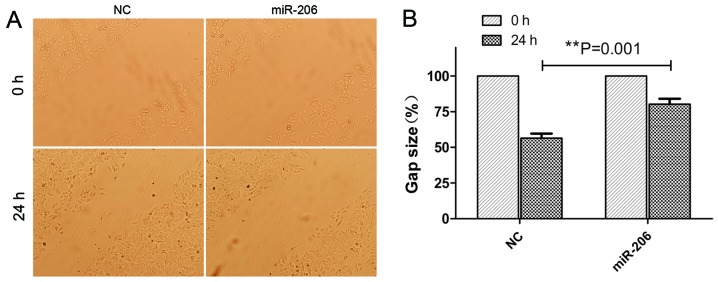
miR-206 inhibits HepG2 cell migration. (A) Wound healing assay was conducted on HepG2 cells transfected with miR-206 or the negatvie contorl (NC). After 24 h, the cells transfected with miR-206 closed the wound more slowly than those transfected with NC. Results from a representative experiment are shown. Magnification, ×100. (B) The residual gap between the migrating cells from the opposing wound edge was expressed as a percentage of the initial gap size. ^**^P<0.01.

Cellular migration is an essential process in cancer metastasis. Thus, we examined the cellular migration ability in order to explore the potential role of miR-206 in HCC cell metastasis. The wound healing assay revealed that the cells transfected with miR-206 healed the wound more slowly than the NC-transfected cells (Fig. 8). MMPs may be associated with the impaired migtation of miR-206-transfected cells. To examine this hypothesis, we detected MMP-9 expression at the mRNA and protein level. Consistent with the results of migration assay, the overexpression of miR-206 caused a significant reduction in MMP-9 expression at the mRNA and protein level (Figs. 3B and 6); the protein expression level of cleaved MMP-9 (MMP-9 CL) in particular was downregulated in the HepG2 cells. Our results indicate that one of the possible mechanims responsible for the inhibitory effects of miR-206 on the migration of HepG2 cells is through the Notch3-MMP-9 pathway, at least through the downregulation of MMP-9.

## Discussion

An abundance of *in vivo* and *in vitro* studies has indicated that enhanced cell proliferation, resistance to apoptosis and the migration state of HCC cells plays an important role in the progression of HCC (2,8). Despite increasing evidence pointing to a role for miR-206 as a tumor suppressor, the tumor suppressive effect of miR-206 has not been fully elucidated. To the best of our knowledge, the present study is the first to explore the function and probable underlying mechanisms of action of miR-206 in HCC HepG2 cells. First, using immunohistochemistry, we found that Notch3 protein expression was markedly increased in the HCC tissues compared with the adjacent normal tissues; these results are consistent with those of previous studies suggesting that the increased expression levels of Notch3 significantly correlates with HCC progression and unfavorable prognosis (19,26,29). Secondly, miR-206 mimic and mimic negative control were successfully transfected into the HepG2 cells. We also found that elevated miR-206 levels inhibited the growth of HepG2 cells, which was associated with the induction of apoptosis and cell cycle arrest. In addition, cellular migration was also impaired following transfection with miR-206 in the HepG2 cells. Our results demonstrated that there are hopeful prospects for miR-206 gene therapy in HCC; however, the possible underlying mechanisms require further investigation.

It has been demonstrated that several target mRNAs are directly regulated by miR-206, including Cdc42, estrogen receptor α (ERα), Notch3, liver X receptor α (LXRα), high mobility group box 3-like pseudogene (Hmgb3) and c-Met (12–13,15,30–32), among which Notch3 was hypothesized to be a direct target gene of miR-206 in this study. Of note, we found that the enforced overexpression of miR-206 markedly attenuated Notch3 expression at the mRNA and protein level in the HepG2 cells, these results are consistent with those of other studies on other cell lines (1,15). Therefore, to a certain extent, this result supports our hypothesis that Notch3 is a direct target gene of miR-206 in HepG2 cells.

An increasing number of studies has suggested that Notch3 has a potential role in anti-apoptosis (1,15,21,25); however, the underlying mechanisms of this anti-apoptotic role remain unclear. Wang *et al* reported that Notch3 signaling can help smooth muscle cells resist Fas ligand-induced apoptosis (33). However, our results revealed that Notch3 was downregulated with the overexpression of miR-206; Bax expression was increased at the mRNA and protein levels, Bcl-2 expression was significantly reduced, and, finally, caspase-3, which exhibited a similar effect to GSI (25), was activated. Thus, our results indicate that the pro-apoptotic effect of miR-206 in HepG2 cells is at least partially dependent on Notch3-mediated mitochondrial apoptotic signaling. However, it has been shown that there are 2 different mechanisms associated with cell apoptosis: the extrinsic receptor-mediated pathway and the intrinsic mitochondrial-dependent pathway (34). Therefore, the anti-apoptotic effects of miR-206 in HCC require further investigation.

Moreover, miR-206 inhibited the growth of HepG2 cells by inducing cell cycle arrest. Our results revealed that the overexpression of miR-206 markedly downregulated Hes1 expression, significantly elevated p57 expression, and finally induced cell cycle G1 phase blockage in the HepG2 cells. These results are in accordance with those of a previous study (27), which reported that p57 is a target of transcriptional repression by the Notch3 effector, Hes1. Of note, the same study also found that the upregulation of p57 by cDNA transfection decreased tumor growth, as demonstrated by the growth curve, flow cytometric analysis and cyclin D1 downregulation, without affecting the apoptotic machinery. Similarly, Chen *et al* found that miR-206 overexpression suppressed ERα and induced the cell cycle arrest of ERα-positive epithelial endometrial cells (EECs) (13). Moreover, it has been demonstrated that estrogens play an important role in the control of liver cell proliferation (35). Thus, the issue of whether miR-206 induces cell cycle arrest in HCC cell lines by inhibiting ERα, remains to be addressed further.

Cell migration and invasion are involved in a number of physiological processes as normal events. However, uncontrolled migration and invasion lead to metastasis, which is the cause of as high as 90% of human cancer-related deaths (36). Metastasis is a multistep process; MMPs are involved in cell migration and invasion and are frequently upregulated in cancer cells (37–39). In the present study, both the mRNA and protein levels of MMP-9 were downregulated in the miR-206-transfected cells, which significantly impaired the migratory capability of HepG2 cells. In a previous study, using Transwell assay, it was demonstrated that 95D cells transfected with miR-206 had a decreased invasive capability than the cells transfected with non-specific control miRNA (14). In the present study, we demonstrated that miR-206 inhibited cell migration through the Notch3-MMP-9 pathway, partly due to its effect on MMP-9.

Although we suggested Notch3 is likely to be a direct target gene of miR-206 in HepG2 cells, further studies are required to identify any other mRNAs that are directly regulated by miR-206 in HepG2 cells, as previously reported in other cell lines (12,13,30–32). In addition, the lack of *in vivo* validation of our molecular pathway in HCC cancer is a limitation of this study. However, to the best of our knowledge, our study is the first to reveal the function and possible underlying mechanisms of action of miR-206 in HCC HepG2 cells.

It is worth noting that the modulation of a target mRNA by several miRNAs and the simultaneous regulation of a variety of mRNAs by a single mRNA is a normal phenomenon (40). Furthermore, Di Leva *et al* suggested a negative transcriptional regulatory loop in which miR-221 and miR-222 target ERα, which, in turn, suppresses miR-221 and miR-222 expression (11). There seems to be a doubt as to whether there is a similar interaction between miR-206 and Notch3. Therefore, further studies are required to elucidate all the aspects of miR-206 expression in HCC.

Recent studies (41,42) have shown that miR-206 expression is significantly downregulated in gastric and breast cancer tissues when compared with their normal adjacent tissues, which significantly correlates with tumor progression, suggesting that miR-206 acts as a tumor suppressor.

**Figure 9:**
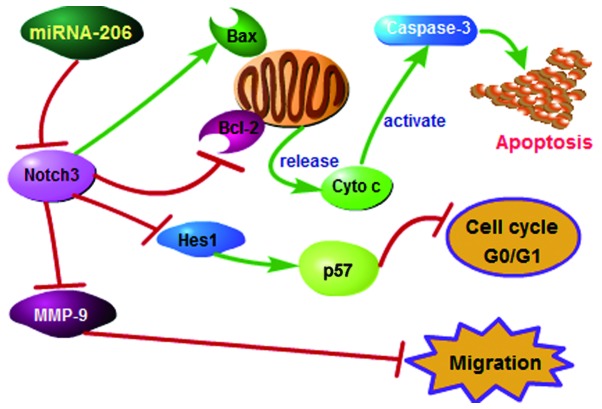
Diagram of the roles of miR-206 in hepatocellular carcinoma (HCC) HepG2 cells. First, miR-206 overexpression downregulated Notch3 expression and, in turn, Bax was expression increased, Bcl-2 expression was reduced, which activated caspase-3 and induced cellular apoptosis through mitochondrial apoptotic signaling. Moreover, the decreased Notch3 expression resulted in the downregulation of Hes1 and the upregulation of p57 expression, and induced cell cycle G1 phase blockage in the HepG2 cells. In addition, miR-206 inhibited cell migration through the Notch3-matrix metalloproteinase-9 (MMP-9) pathway.

Taken together, our results demonstrate that miRNA-206 overexpression promotes apoptosis, induces cell cycle arrest and inhibits the migration of HCC HepG2 cells. The possible underlying mechanisms of action of miR-206 in HCC are shown in Fig. 9. In conclusion, this study suggests that the delivery of miR-206 to HepG2 cells may lead to the development of novel therapeutic strageties for HCC, and that miR-206 may be a potential therapeutic agent for human tumors, and is worthy of further investigation.

## Figures and Tables

**Table I:** Primers used for qRT-PCR.

Gene symbol	NCBI RefSeq no.	Sequence (5′→3′)		Product length (bp)
Notch3	NM_000435	(F) GTGTGTGTCAATGGCTGGAC	(R) GTGACACAGGAGGCCAGTCT	150
Bax	NM_138763	(F) CCCGAGAGGTCTTTTTCCGAG	(R) CCAGCCCATGATGGTTCTGAT	155
Bcl-2	NM_000633	(F) CTTTGAGTTCGGTGGGGTCA	(R) GGGCCGTACAGTTCCACAAA	162
Hes1	NM_005524	(F) TCAACACGACACCGGATAAAC	(R) GCCGCGAGCTATCTTTCTTCA	153
p57	NM_000076	(F) CCCTTCTTCTCGCTGTCCTC	(R) CTGGTCCTCGGCGTTCA	231
MMP-9	NM_004994	(F) CTGCAGTGCCCTGAGGACTA	(R) ACTCCTCCCTTTCCTCCAGA	135
β-actin	NM_001101	(F) TTAGTTGCGTTACACCCTTTC	(R) GCTGTCACCTTCACCGTTC	156
